# The deubiquitinase USP10 protects pancreatic cancer cells from endoplasmic reticulum stress

**DOI:** 10.1038/s41698-022-00336-x

**Published:** 2022-12-21

**Authors:** Udayan Bhattacharya, Elangovan Thavathiru, Fiifi Neizer-Ashun, Chao Xu, Zoran Gatalica, Shailendra Kumar Dhar Dwivedi, Anindya Dey, Priyabrata Mukherjee, Resham Bhattacharya

**Affiliations:** 1 Department of Obstetrics and Gynecology, University of Oklahoma Health Sciences Center, Oklahoma City, OK USA; 2 Department of Cell Biology, University of Oklahoma Health Sciences Center, Oklahoma City, OK USA; 3 Department of Biostatistics and Epidemiology, University of Oklahoma Health Sciences Center, Oklahoma City, OK USA; 4 Department of Pathology, University of Oklahoma Health Sciences Center, Oklahoma City, OK USA; 5 Peggy and Charles Stephenson Cancer Center, University of Oklahoma Health Sciences Center, Oklahoma City, OK USA

**Keywords:** Cancer, Protein folding

## Abstract

The ubiquitin-specific peptidase 10 (USP10) plays a context-specific, pro or anti-tumorigenic role in different malignancies. However, the role of USP10 in pancreatic cancer remains unclear. Our protein and RNA level analysis from archived specimens and public databases show that USP10 is overexpressed in pancreatic ductal adenocarcinoma (PDAC) and expression correlates with poor overall patient survival. Phenotypically, silencing USP10 decreased viability, clonal growth and invasive properties of pancreatic cancer cells. Mechanistically, silencing USP10 upregulated BiP and induced endoplasmic reticulum (ER) stress that led to an unfolded protein response (UPR) and upregulation of PERK, IRE1α. Decreased cell viability of USP10 silenced cells could be rescued by a chemical chaperone that promotes protein folding. Our studies suggest that USP10 by protecting pancreatic cancer cells from ER stress may support tumor progression.

## Introduction

Pancreatic cancer is the third leading cause of cancer-related mortality^1,2^ with pancreatic ductal adenocarcinoma (PDAC) constituting 90% of all pancreatic cancer cases. PDAC has an average 5-year survival rate of less than 10% because most patients have advanced and unresectable disease at the time of diagnosis. Moreover, current cytotoxic therapies are not very effective, therefore, a better understanding of the molecular mechanisms driving this cancer is warranted to design improved therapeutics.

In this context, the ubiquitin specific peptidase 10 (USP10) has been shown to be upregulated in several malignancies. USP10 is a member of the USP family with established roles in cellular and metabolic processes. Notably, USP10 deubiquitinates and stabilizes p53 following DNA damage^3^, promotes the activation of AMPK under energy stress^4^, promotes autophagy^5^, and stabilizes the NOTCH1 intracellular domain (NICD1) in endothelial cells during angiogenic sprouting. The role of USP10 in tumorigenesis is however context dependent. In malignancies such as gastric carcinomas, hepatocellular carcinoma, and intestinal adenocarcinomas, USP10 is suggested to play a tumor suppressing role. While in other malignancies such as prostate cancer, breast cancer, and acute myeloid leukemia, USP10 promotes oncogenic function^6^. Interestingly, the role of USP10 in PDAC has remained largely unexplored.

Here we describe a tumor supportive role for USP10 in pancreatic cancer. We find that compared to the normal or malignant stromal tissue, the expression of USP10 is highest in ductal pancreatic cancer tissues. Analysis of public databases showed that higher expression of USP10 was significantly associated with poorer overall survival of PDAC patients. Depleting USP10 significantly inhibited clonal growth potential and cellular invasion of pancreatic cancer cells. At the molecular level, depleting USP10 although altered ubiquitination of certain ribosomal proteins, however, global protein synthesis was unaffected. Finally, silencing USP10 caused an unfolded protein response (UPR) arising from ER stress that led to decreased cell viability and could be rescued by a chemical chaperone that promotes protein folding. Overall, our studies suggest that USP10 may be a clinically relevant target in pancreatic cancer.

## Results

### Expression and pathological significance of USP10 in pancreatic cancer

**Fig. 1:**
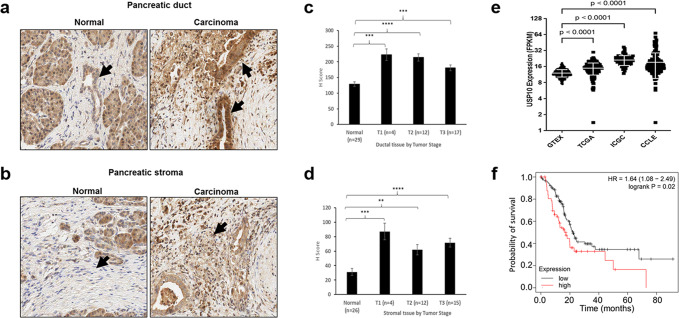
Expression and significance of USP10 in pancreatic cancer. (**a**–**d**) Expression of USP10 in pancreatic tissues: Normal and Pancreatic cancer patients (Ductal tissues: Normal, *n* = 29; cancerous T1 *n* = 4; T2 *n* = 12, T3 *n* = 17 & Stromal tissues: normal (*n* = 26) and cancerous, T1 *n* = 4; T2 *n* = 12; T3 *n* = 15) stained by immunohistochemistry (IHC) to assess the expression levels of USP10 protein. Data were analyzed by Linear Mixed Model. The H-score for ductal tissue in normal sample is estimated to be 93.95, 85.49, and 52.77 units less than the H-score in T1, T2, and T3 sample respectively (*p*-value 0.0001, <0.0001, and 0.0001). There is no significant difference between pairwise comparisons of T1, T2, and T3. Similarly, for stroma, the H-score in normal sample is estimated to be 55.90, 30.76, and 40.51 units less than the H-score in T1, T2, and T3 sample respectively (*p*-value 0.0002, 0.0023, and <0.0001). There is no significant difference between pairwise comparisons of T1, T2, and T3. **e** Levels of USP10 mRNA in normal pancreas (GTEx), pancreatic cancer cell lines (CCLE) and pancreatic cancer tissues (ICGC and TCGA). Data represent mean ± SD. Using Kruskal-Wallis rank sum test, the FPKM/expression was significantly higher in TCGA, ICGC, and CCLE compared to GTEx (*p*-value <2.2e−16). **f** Kaplan-Meier overall survival curves in USP10 low and high expression pancreatic cancer cases (*p* = 0.02). The proportion survival is plotted versus time (since diagnosis in month). Kaplan-Meier curves with a log-rank test where *P* value < 0.05 is considered significant.

To evaluate the expression of USP10, pancreatic ductal adenocarcinoma and adjacent normal pancreatic tissues from thirty-three previously archived specimens were analyzed by immunohistochemistry. Standardized H score was derived from the intensity of staining and the percentage of cells staining positively. While expression of USP10 was low in normal pancreatic tissues, overall expression was higher in the malignant ductal tissues compared to the stroma (Fig. 1a, b). Staining for USP10 was predominantly cytoplasmic, and was high in the ductal epithelium, T1 through T3 stages, compared to the normal pancreatic epithelium (Fig. 1a, c). Similarly, malignant stroma of T1 through T3 stages showed higher staining than their normal counterparts (Fig. 1b, d). These results suggest that USP10 may play a role in pancreatic tumor growth.

We next investigated the clinical relevance of USP10 expression in pancreatic cancer. We used publicly available annotated databases for comparison of RNA Seq data from Genotype-Tissue Expression (GTEx) for normal pancreas, Cancer Cell Lines Encyclopedia (CCLE) for pancreatic cancer cell lines, and the International Cancer Genome Consortium (ICGC), and the Cancer Genome Atlas (TCGA) for pancreatic cancer tissue level expression. Compared to the normal pancreas (GTEx, mean = ~12), the expression of USP10 was higher in the pancreatic cancer cell lines (mean = ~32), and in the pancreatic cancer patient samples (mean for ICGC = ~21, for TCGA = ~15) (Fig. 1e).

Analyzing the clinically annotated TCGA database we noted that higher expression of USP10 was significantly associated (log-rank test *p* = 0.02) with poorer overall survival of PDAC patients, with a hazard ratio of 1.64. Median survival of the low-expression group was about 22 months, compared to 16 months for the USP10 high-expression group (Fig. 1f). Together these results support a role for USP10 in the pathology of pancreatic cancer and we next sought to determine its functional significance.

### Role of USP10 in regulating cancer phenotypes

**Fig. 2:**
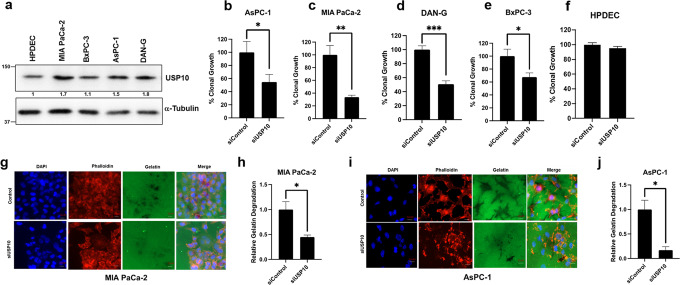
Cell line expression and cellular phenotypes of USP10 silencing. **a** Immunoblot showing expression of USP10 in pancreatic cancer cell lines along with normal HPDEC cells. The numbers denote densitometric quantitation of USP10, normalized with respect to α-Tubulin by NIH Image J. **b**–**e** Anchorage-independent clonal potential evaluated in control and USP10 silenced pancreatic cancer cell lines and HPDEC. Percent clonal growth along with SD is plotted and compared to their respective siCTL which was set to 100%. Student’s *t*-test was performed for statistical analysis and *P* < 0.05 was considered significant; Experiments were repeated independently at least 3 times. (**g**–**j**) AsPc-1 and MiaPaCa-2 cells were transfected with either non-target control siRNA (siCTL) or siRNA targeting USP10 (siUSP10). 72 h post-transfection, cells were fixed and stained with DAPI (blue), FITC-gelatin (green), and phalloidin (Tubulin, red). Relative FITC-gelatin matrix degradation i.e., ability to migrate and invade, in siCTL and siUSP10 transfected cells is quantitated; Cells that showed FITC-gelatin degradation were counted and this was set to 1 in the control group. Similarly, in the USP10 silenced group, cells that showed gelatin degradation were counted and expressed relative to the control; >100 cells were evaluated, Data represent mean ± SD; Student’s *t*-test was performed for statistical analysis and *P value < 0.05. (Scale Bar = 10 μm).

We first determined the expression levels of USP10 in different pancreatic cancer cell lines and in the non-malignant human pancreatic ductal epithelial cells (HPDEC) by immunoblotting. Compared to HPDEC, the K-Ras wild-type BxPC-3 cells expressed USP10 at similar levels, while the expression was ~1.7, ~1.5, and ~1.8 fold significantly higher in the K-Ras mutated MIA PaCa-2, AsPC-1, and DAN-G cells respectively (Fig. 2a). These results are in accordance with RNA level expression data obtained from the CCLE database as depicted in Fig. 1a.

Intriguingly, a cancer-context dependent, tumor promoting or tumor suppressing function has been attributed to USP10^7^. However, higher USP10 expression is associated with poorer survival therefore, we assessed its contribution towards pancreatic cancer growth and cellular invasion using the clonal growth and the FITC-based gelatin degradation assays respectively. Interestingly, siRNA-mediated down-regulation of USP10 in the pancreatic cancer cell lines significantly decreased clonal growth by ~32–66%, while the non-malignant HPDEC showed no effect (Fig. 2b, f, Supplementary Fig 1). Interestingly, the K-Ras wild-type BxPC-3 cells also showed a ~32% decrease in clonal growth (Fig. 2e), suggesting that the impact of USP10 depletion in pancreatic cancer cells is independent of the K-Ras status. Next, we performed the FITC-gelatin matrix degradation assay that allows quantitation of the invasive potential of cancer cells. Depletion of USP10 significantly decreased matrix degradation by ~0.6 fold in MIA PaCa-2 cells (Fig. 2g, h) and by ~0.8 fold in AsPC-1 cells respectively (Fig. 2i, j). Thus, silencing USP10 inhibits clonal growth and invasion in pancreatic cancer cell lines, two pivotal hallmarks of cancer progression.

### USP10 silencing induces an unfolded protein response

**Fig. 3:**
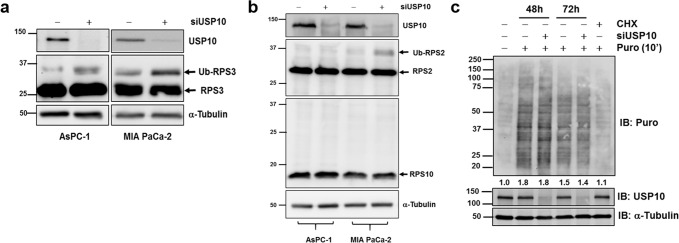
USP10 deubiquitinates ribosomal subunits. **a**, **b** Monoubiquitination changes of RPS2, RPS3, and RPS10 after USP10 depletion was evaluated by immunoblotting. α Tubulin was used as loading control and efficient USP10 inhibition was confirmed by immunoblotting. **c** Control and USP10 silenced cells were incubated with 10 μg/ml of puromycin 48 h or 72 h post-transfection for 10 min prior to harvest. For negative control, cells were treated with cycloheximide (30 μg/ml, 60 min) prior to puromycin incubation. Global protein synthesis was evaluated by anti-puromycin antibody. The numbers denote densitometric quantitation of individual lanes, normalized with respect to α-Tubulin by NIH Image J.

We then sought to delineate USP10 signaling at the molecular level that could potentially explain the observed phenotypic effects. Recently, the G3BP1-family-USP10 complexes that are components of the cytoplasmic stress granules have been implicated in ribosomal quality control^8^. Ribosomes stalled in translation undergo site-specific deubiquitination of the 40S ribosomal subunits, RPS2, RPS3, and RPS10 by the G3BP1-family-USP10 complexes rescuing or preventing the 40S subunit from programmed lysosomal degradation. Therefore, we considered that down-regulation of USP10 may disrupt recycling of ribosomal subunits hence affecting overall protein synthesis. In this context, we performed immunoblotting for total and ubiquitinated RPS3, RPS2, and RPS10 in the K-Ras mutated AsPC-1 and MIA PaCa-2 cell lines. In USP10 silenced cells, we observed a consistent increase in monoubiquitinated RPS3 in both AsPC-1 and in MIA PaCa-2 cells (Fig. 3a). RPS2 monoubiquitination was also observed, however only in MIA PaCa-2 cells (Fig. 3b), while ubiquitination of RPS10 was not observed (Fig. 3b), suggesting that while USP10 may be important for deubiquitinating critical 40S subunits like RPS3, it may not be essential for modifying other subunits which may require additional factors. We then utilized the surface sensing of translation (SunSET) assay^9^, to evaluate the protein synthesis. This method relies on the principle that puromycin, an amino-nucleoside antibiotic, because of its structural analogy to tyrosyl t-RNA, is incorporated into the elongating peptide chains. The puromycin incorporated peptides can then be detected by immunoblotting with an anti-puromycin antibody, which represents the overall rate of protein synthesis. We did not observe any major differences in the protein synthesis rate in MIA PaCa-2 cells despite robust silencing of USP10 at 48 or 72 h (Fig. 3c). As expected, the negative control or the positive control samples with cycloheximide (CHX) treatment did not incorporate puromycin (Fig. 3c).

**Fig. 4:**
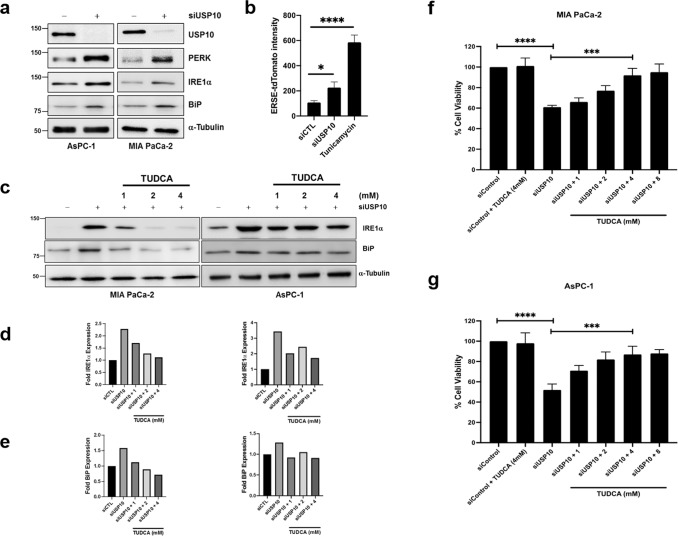
Silencing USP10 induces ER stress and unfolded protein response. **a** Immunoblotting for markers of UPR after 72 h USP10 silencing. **b** Quantitation of fluorescent intensity of BiP-ERSE-TdTomato reporter in siCTL, siUSP10, and tunicamycin treated cells. **c** AsPc-1 and MiaPaCa-2 were transfected with control or USP10 siRNA and treated with indicated doses of TUDCA for 48 h and evaluated for UPR markers by immunoblotting (**d**, **e**) and quantified by densitometry analysis using Image J and normalized to their respective α-tubulin levels and then compared to siCTL which was set to 1 (**f**, **g**) Quantitation of cell viability after similar treatments in **c** by MTS. Data represent mean ± SD; Ordinary one-way ANOVA was performed for statistical analysis and ****P < 0.001 and ****P < 0.0001. A post-hoc Tukey test was performed for comparisons between the groups. Experiments were repeated independently at least 3 times.

Next, we asked whether the protein folding capacity of pancreatic cancer cells was impaired after USP10 silencing even though global protein synthesis was unaffected. We first immunoblotted for markers of the unfolded protein response (UPR) pathway in control and in USP10 silenced MIA PaCa-2 and AsPC-1 cells. Activation of UPR serves as a buffer for protein folding stress at the endoplasmic reticulum (ER). Interestingly, we observed augmented expression of PERK, IRE1α, and BiP in USP10 silenced cells (Fig. 4a), suggestive of ER stress and subsequent UPR induction. To further validate ER stress induction after USP10 silencing, we analyzed the expression of a fluorescent reporter under the control of ER stress response element (ERSE) of the BiP promoter (BiP-ERSE-TdTomato)^10^ in MIA PaCa-2 cells. We observed that expression of the reporter, as measured by mean fluorescent intensity was induced by tunicamycin treatment and by silencing USP10, compared to control (Fig. 4b), corroborating ER stress and UPR induction upon USP10 depletion. We then determined whether treatment of USP10 silenced cells with tauroursodeoxycholic acid (TUDCA) would relieve ER stress and support cellular viability. TUDCA is a bile acid derivative that acts as chemical chaperone to enhance protein folding and protect cells against ER stress^11^. Treatment with TUDCA normalized expression of IRE1α and BiP to near control levels (Fig. 4c–e). Silencing USP10 alone decreased cell viability by ~40% in MIA PaCa-2 cells and by ~48% in AsPC-1 cells. Interestingly, TUDCA treatment rescued the growth-suppressive effect of USP10 silencing in a dose-dependent manner in both cell lines (Fig. 4f, g). Compared to their normal counterpart, cancer cells have an increased demand for protein synthesis and are subject to various metabolic and redox stress which necessitate a heightened protein folding response that can buffer ER stress. Altogether, our results suggest that USP10 may be required for modulating the increased demand for protein folding ability, especially in cancer cells, and prevent potentiation of ER stress.

## Discussion

We have identified the ubiquitin-specific peptidase 10 (USP10) to play a pro-tumorigenic role in pancreatic cancer. Several pro and anti-tumorigenic roles of USP10 have been reported in different malignancies. In colon, gastric, and lung cancer tissues, USP10 levels are decreased compared to normal tissue^12–14^. In these malignancies, USP10 was shown to inhibit c-Myc transcription, and AKT/mTOR activation to inhibit cancer cell growth^12,15,16^. Conversely, USP10 is overexpressed in glioblastoma multiforme, prostate, and breast cancers^17–19^. Here, we show that USP10 is overexpressed in PDAC patient tissues and K-Ras mutated pancreatic cancer cell lines and higher expression of USP10 was significantly associated with poor overall survival of PDAC patients. USP10 was overexpressed in both malignant ductal and stromal tissues. Interestingly, we found comparable expression in PDAC tissues regardless of tumor stage, suggesting that the oncogenic functions of USP10 may be required early and through the course of disease progression. Furthermore, metastasis is an inherent attribute of many types of cancer including pancreatic cancer. Degradation of the extracellular matrix (ECM) by cancer cells is a prerequisite for invasion and migration to distant sites. We show that USP10 depletion inhibited cellular invasion in PDAC cells and decreased clonogenic ability, suggesting that silencing USP10 may decrease metastatic potential of pancreatic cancer.

USP10 has been shown to be important for deubiquitinating ribosomal proteins i.e RPS2, RPS3, RPS10 to facilitate recycling and prevent degradation of ribosomal subunits^8,20^. While we did not observe ubiquitination of RPS10, we found an increase in monoubiquitinated RPS3 in both AsPc-1 and in MiaPaCa-2 cells and in monoubiquitination of RPS2 in MiaPaCa-2 cells, albeit no change in global protein synthesis after USP10 knockdown. Impairment of ribosomal recycling is associated with mistranslation of proteins and can cause an unfolded protein response^21,22^. Interestingly, we found that silencing USP10 increases ER stress and associated UPR pathways, IRE1 and PERK, which were reversed after treatment with the chemical chaperone, TUDCA. Our results suggest that USP10 may be important for modulating recycling of ribosomal subunits and ER stress protection required to indulge the proteomic necessities of pancreatic cancer cells. Collectively, our study provides new insights into the tumor promoting role of USP10.

## Methods

### Cell culture and chemicals

The human pancreatic cancer cell lines PANC-1 (CRL-14690), AsPC-1 (CRL-1682), MIA PaCa-2 (CRL-1420), and BxPC-3 (CRL-1687) were purchased from American Type Culture Collection, and DAN-G was obtained from German Collection of Microorganisms and Cell Cultures (DSMZ, Braunschweig, Germany). The cells were grown in a 5% CO2 humidified atmosphere in DMEM (10-013-CV, Corning, NY) + 10% FBS (16000-044, Life technologies, Carlsbad, CA, USA), RPMI1640 (10-040-CV, Corning) + 10%FBS. MiaPaCa was cultured in DMEM + 10% FBS + 2.5% Horse serum (R55075, Life technologies). 1% Penicillin-Streptomycin (15140-122, Life technologies) was added to the various culture media.

Non-targeting SiRNA (SIC001) and SiRNA against USP10 (SASI_Hs01_00213007; SASI_Hs01_00213008) were obtained from Sigma-Aldirch. Cycloheximide (C7698), Puromycin (P8833), and Tauroursodeoxycholic acid (TUDCA) (580549) were obtained from Sigma-Aldrich.

### SiRNA transfection

Lipofectamine RNAiMAX (Life Technologies) was used to transfect USP10 SiRNA (50 nM) MiaPaCa-2, HPDEC, BxPC3, and DAN-G, and Dharmafect-2 (Horizon Discovery) was used to transfect the USP10 SiRNA (100 nM) into AsPc-1 as per the manufacturer’s protocols.

### Immunohistochemistry

Immunohistochemistry for USP10 expression in pancreatic cancer was performed on patient samples that were collected at the University of Oklahoma Health Sciences Center. Written informed consent was obtained from all the patients enrolled into the study and Institutional Review Board approval was provided by University of Oklahoma Health Sciences Center. IHC was performed using anti-USP10 (1:500) antibody and the staining intensity of USP10 was scored using the standardized H-score, which takes into account both intensity and percentage of cells stained. The difference in H-score between groups were assessed by Linear Mixed Model.

### Puromycin incorporation assay

Puromycin incorporation during protein synthesis was assessed by modification of a previously described method^9^. Briefly, the siRNA transfected cells were incubated with 10 μg/ml of puromycin for 10 minutes prior to harvest. For negative control, the cells were treated with cycloheximide at 30 μg/ml for 60 minutes prior to adding the puromycin.

### Cell Lysis and Immunoblotting

Total cellular lysate was prepared in RIPA (Boston Bioproducts) and protein concentration was measured using a BCA Assay Kit (Pierce, 23225). A standard protocol was used for immunoblotting. The cell lysates were separated on 10% or 15% glycine SDS-PAGE gel and transferred to PVDF membrane. Membranes were blocked in 5% BSA in TBS with 0.1% TWEEN-20 (TBST) for 1 h at room temperature. Primary antibodies were prepared in TBST with 5% BSA and membranes were incubated in primary antibody at 4 °C for overnight. Antibodies were purchased from Cell Signaling Technologies (USP10, 5553, 1:1000; PERK, 5683, 1:1000; IRE1, 3294, 1:1000; BiP, 3177, 1:1000), Abcam (USP10, Ab72486, 1:500 for immunohistochemistry), ProteinTech (anti-Tubulin-alpha, 66031-1-Ig, 1:10000). Sigma-Aldrich (Anti-puromycin, MABE343, 1:25000), and HRP-conjugated secondary antibodies (anti-mouse and anti-rabbit). Secondary antibodies were used at a concentration of 1:10,000. Unprocessed and uncropped blot scans can be found in the Supplementary Information (Supplementary Figs. 2–5).

### Colony formation assay

SiRNA-transfected cells were harvested 24-hour post-transfection and the cells (200 cells/well) were seeded into 6-well plates in complete growth medium and incubated for another 7–10 days after which the colonies were washed with PBS (02-0119-0500, VWR, Radnor, PA, USA) and then stained with crystal violet solution (20% alcohol (v/v) + 0.1% crystal violet (w/v) (B21932, Alfa Aesar, Haverhill, MA, USA)). After washing with water, the colonies were dried and counted with the GelCount (Oxford Optronix, Abingdon, UK).

### Gelatin degradation assay

Acid-washed coverslips were first coated with 50 μg/mL poly-L-lysine for 20 minutes at room temperature, and then fixed with 0.5% glutaraldehyde for 15 minutes. Gelatin matrix was prepared by mixing 0.2% gelatin and Oregon Green 488 Gelatin Conjugate (Life Technologies) at a 7:1 ratio. After coating for 10 minutes, coverslips were washed with PBS and self-fluorescence was quenched with 5 mg/mL sodium borohydride for 15 minutes followed by washing with PBS. For degradation assay, 10^5^ MIA PaCa-2 cells or 1.5 × 10^5^ AsPC-1 cells seeded in each well of a 12-well plate containing 488 Gelatin Conjugate cover slips. 12 or 48 h after plating, cells were fixed in 4% paraformaldehyde and stained with Alexa Flour 568 Phalloidin (Life Technologies) for 15 minutes at room temperature. The cells were washed with PBS and mounted with VECTASHIELD-mounting medium containing DAPI (Vector Laboratories). Images were acquired at 40x using the Zeiss Axio-Observer Z1. Cells that degraded the extracellular matrix at focal adhesions sites were scored positive and approximately 200 random cells were quantified. The percentage of cells showing degradation was plotted.

### Reporting summary

Further information on research design is available in the Nature Research Reporting Summary linked to this article.

## Supplementary information

Supplementary Data

REPORTING SUMMARY

## Data Availability

RNA expression data were obtained from publicly available databases, TCGA, CCLE, ICGC, and GTEx. Immunohistochemistry data are not publicly available to protect patient privacy, but will be made available to authorized researchers who have an approved Institutional Review Board application. Please contact the corresponding author with data access requests. All other datasets generated during the study will be made available upon reasonable request to the corresponding author, Dr. Resham Bhattacharya, email address: resham-bhattacharya@ouhsc.edu.

## Code availability

No custom code or scripts were created to perform the data analyses in this study. Graphs were made using GraphPad Prism (v8).
