# Supplementary material for: The deubiquitinase USP10 protects pancreatic cancer cells from endoplasmic reticulum stress
Source: NPJ Precis Oncol. 2022 Dec 21;6:93. doi: 10.1038/s41698-022-00336-x (PMC9772324; doi:10.1038/s41698-022-00336-x)
Supplement: Supplementary file 1 — Supplementary Data [file 41698_2022_336_MOESM1_ESM.pdf]

Supplementary Figure 1

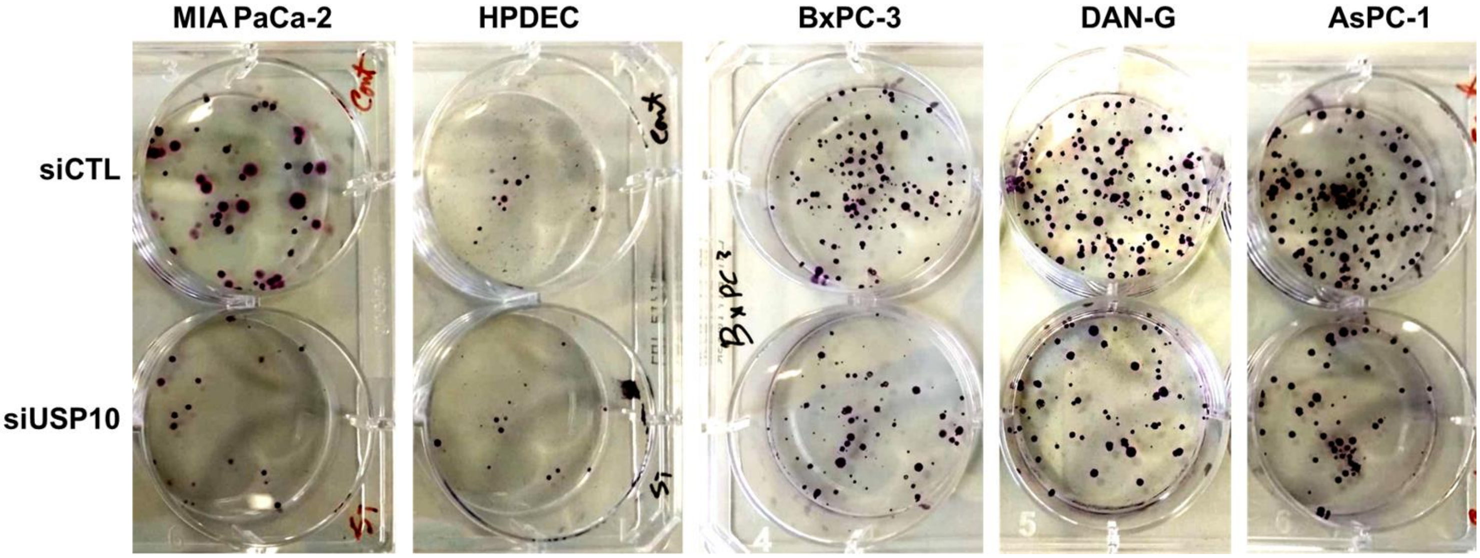

Figure 2. Silencing USP10 decreases clonal growth

Representative images of anchorage independent clonal potential evaluated in control and USP10 silenced pancreatic cancer cell lines and HPDEC.

Supplementary Figure 2

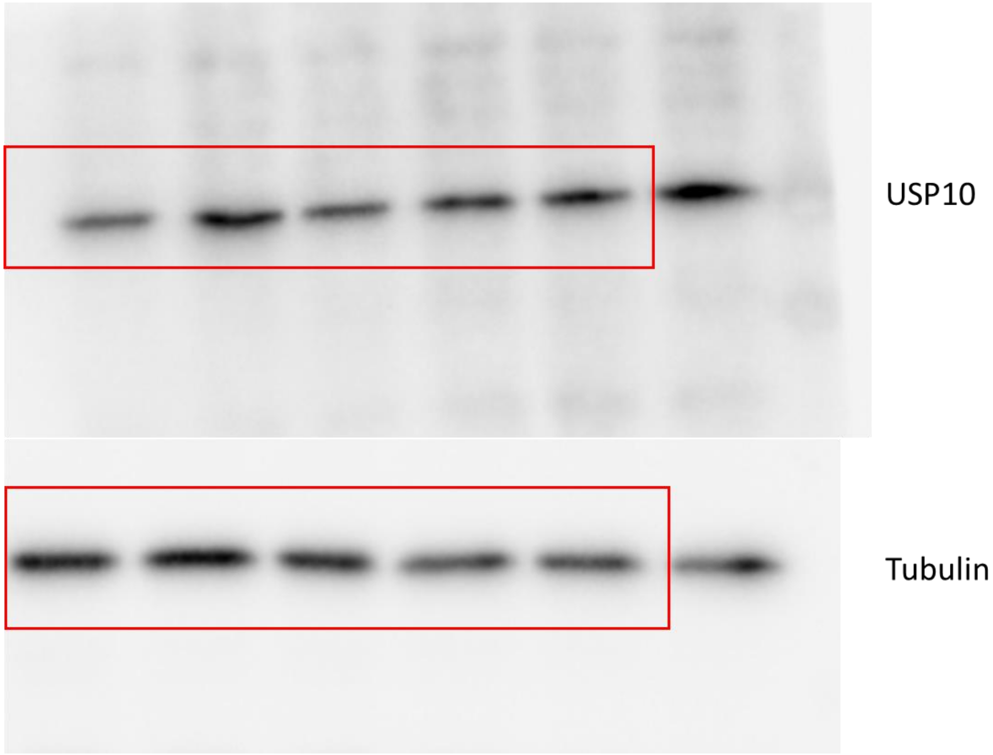

Uncropped Blots for Figure 2a

Supplementary Figure 3

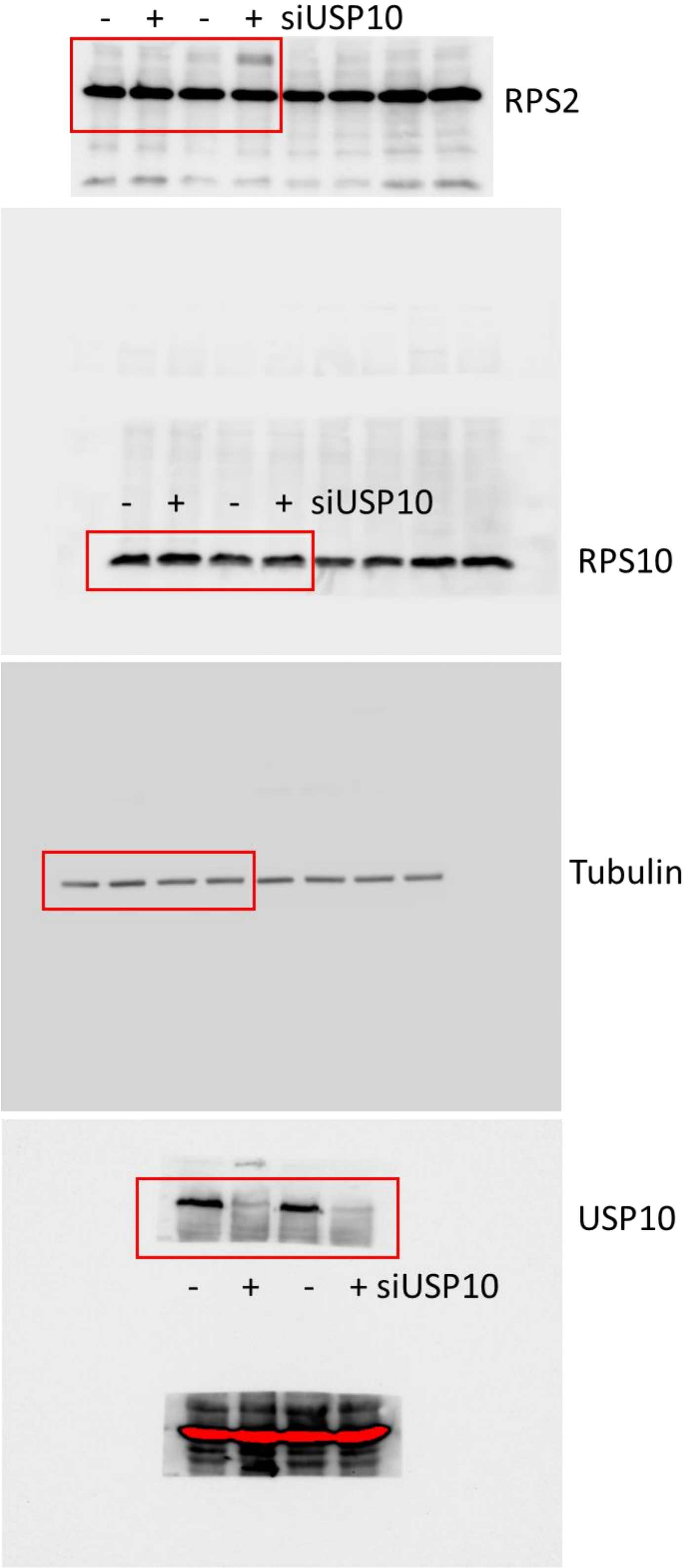

Uncropped Blots for Figure 3b

Supplementary Figure 4

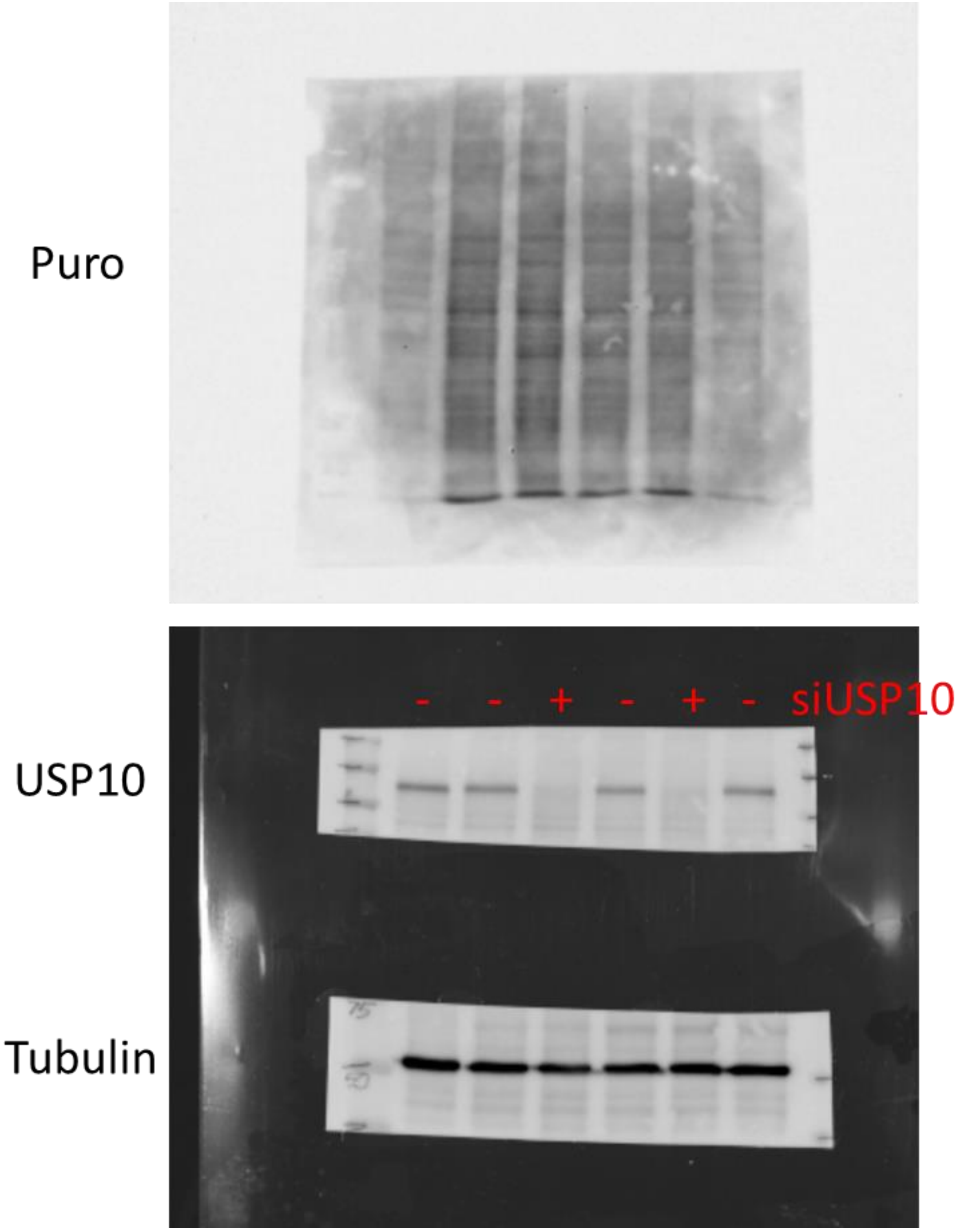

Uncropped Blots for Figure 3c

Supplementary Figure 5

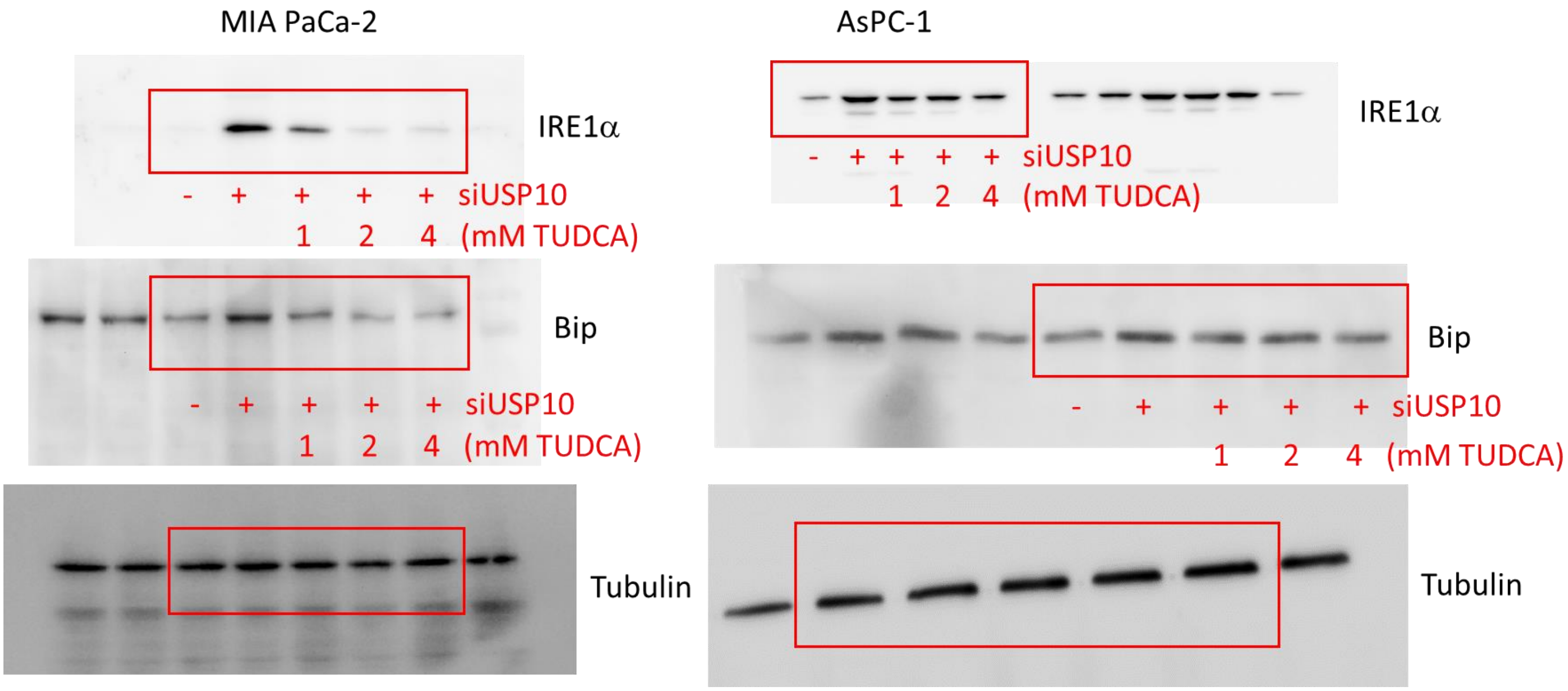

Uncropped Blots for Figure 4c
